# Chiari Malformation Type I: A Review of Pathophysiology, Cerebrospinal Fluid Flow Dynamics, Diagnosis, Surgical Management, and Its Relationship to Syringomyelia

**DOI:** 10.7759/cureus.101226

**Published:** 2026-01-10

**Authors:** Maryam S Alhosani, Sopio Gachechiladze

**Affiliations:** 1 Department of Medicine, University of Georgia, Tbilisi, GEO

**Keywords:** cerebellar tonsillar herniation, chiari malformation type i, csf dynamics, csf flow obstruction, hydrocephalus, neurological outcome, posterior fossa decompression, syringomyelia

## Abstract

Chiari malformation type I (CM-I) is a neurological disorder characterized by the herniation of the cerebellar tonsils through the foramen magnum, resulting in the obstruction of cerebrospinal fluid (CSF) flow and the compression of the brainstem and upper cervical spinal cord. These disturbances contribute to symptoms such as headaches exacerbated by Valsalva maneuvers, dizziness, neck pain, muscle weakness, sensory disturbances, and coordination difficulties. This review provides an overview of the pathophysiology of CM-I, the role of disrupted CSF dynamics, and diagnostic approaches including magnetic resonance imaging (MRI) and cine MRI. Moreover, this review provides current management strategies ranging from conservative therapy to posterior fossa decompression. Additionally, the relationship between CM-I and syringomyelia is examined, emphasizing how altered CSF flow contributes to syrinx formation. Greater understanding of these mechanisms supports earlier diagnosis and more targeted treatment, highlighting the need for continued research into neuroprotective therapies that enhance long-term outcomes and quality of life for patients with CM-I.

## Introduction and background

**Figure 1:**
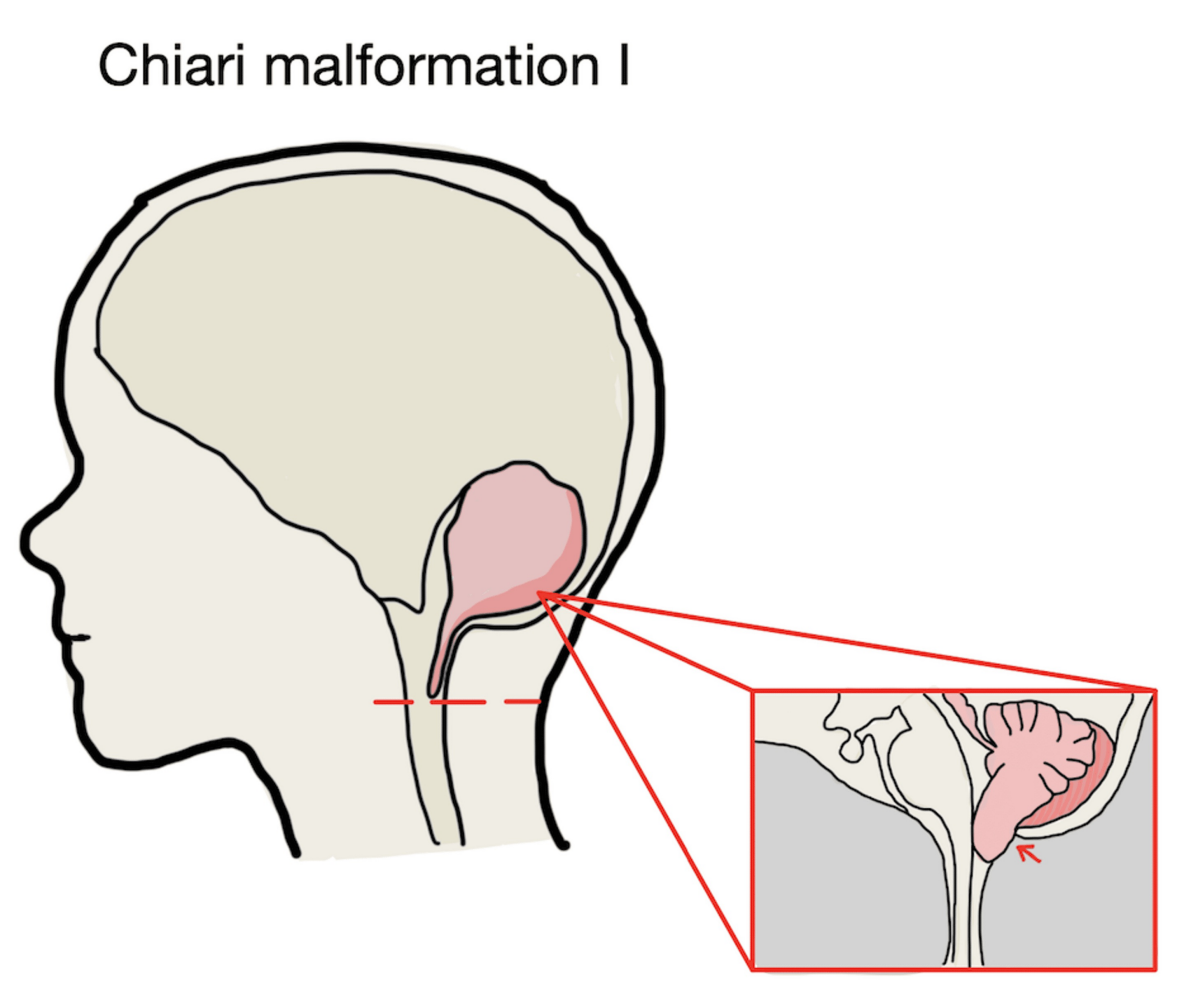
Schematic illustration of CM-I demonstrating the inferior descent of the cerebellar tonsils through the foramen magnum, resulting in hindbrain crowding and CSF flow obstruction The depiction is based on established diagnostic criteria defining CM-I as tonsillar herniation ≥5 mm below the foramen magnum [1,2]. CM-I: Chiari malformation type I; CSF: cerebrospinal fluid

Chiari malformation type I (CM-I) is a congenital or acquired structural abnormality characterized by the caudal displacement of the cerebellar tonsils through the foramen magnum by ≥5 mm [1,2]. This downward herniation disrupts the normal craniospinal pressure gradient and leads to the obstruction of cerebrospinal fluid (CSF) flow at the craniocervical junction. As a result, patients may develop increased intracranial pressure, compression of the brainstem and upper cervical spinal cord, and formation of syringomyelia due to altered CSF pulsatility within the spinal subarachnoid space. Figure 1 illustrates the anatomical changes associated with CM-I, including inferior displacement of the cerebellar tonsils and alteration of CSF pathways at the craniovertebral junction.

Epidemiologically, CM-I is the most common Chiari malformation. This malformation type is estimated to occur in approximately one in 1000 live births. CM-I is increasingly recognized due to the widespread use of magnetic resonance imaging (MRI), with prevalence estimated at 1% in the general population. Although some individuals remain asymptomatic, many present with hallmark symptoms such as occipital headaches exacerbated by coughing, sneezing, or straining (Valsalva maneuvers), neck pain, sensory disturbances, dizziness, imbalance, or cranial nerve dysfunction. In pediatric and adolescent patients, scoliosis may be a presenting feature, often associated with syringomyelia [2-4].

Given the heterogeneity of symptoms and overlap with other neurological disorders, accurate diagnosis and understanding of underlying pathophysiological mechanisms, including CSF flow obstruction and syrinx formation, are critical for optimal management. Posterior fossa decompression remains the primary surgical intervention for symptomatic CM-I, aiming to restore CSF circulation, relieve neural compression, and reduce syrinx size when present.

This review summarizes the current knowledge on CM-I, with emphasis on pathophysiology, CSF flow dynamics, clinical presentation, diagnostic evaluation, and contemporary surgical approaches. Additionally, it highlights the strong relationship between CM-I and syringomyelia and underscores the importance of early recognition and individualized treatment strategies.

## Review

Methods

This narrative review was conducted using peer-reviewed literature obtained from PubMed, Google Scholar, and major neurosurgical and radiology textbooks published between 1994 and 2025. Search terms included "Chiari malformation type I", "cerebellar tonsillar herniation", "CSF flow dynamics", "syringomyelia", and "posterior fossa decompression". Articles discussing epidemiology, pathophysiology, clinical presentation, imaging diagnostics, management options, and postoperative outcomes were included. Non-English publications, isolated case reports without clinical relevance, and non-peer-reviewed sources were excluded. This review synthesizes current evidence to highlight key diagnostic features, treatment strategies, and long-term outcomes in individuals with CM-I.

Pathogenesis (etiology)/embryology and posterior fossa development

The embryological and pathophysiological mechanisms underlying Chiari malformations have been reported with several theories proposed to explain posterior fossa underdevelopment, hindbrain herniation, and altered CSF dynamics. Comprehensive reviews have also highlighted the interplay between abnormal embryologic development, craniocervical crowding, and pressure dissociation across the foramen magnum as central contributors to disease manifestation and associated conditions such as syringomyelia. The pathophysiological sequence leading to hindbrain herniation and CSF obstruction is illustrated schematically below. 

The causes of Chiari malformation vary and may be congenital or acquired. In many individuals, Chiari malformation develops when the lower back part of the brain (cerebellar tonsils) pushes through the foramen magnum, where the brain and spinal cord meet. This often occurs when the posterior fossa is smaller than expected, causing the cerebellum to sit lower than normal and become crowded in the area where the cerebellum and spinal cord should be located. This increased pressure leads to cerebellar tonsillar displacement and downward herniation through the foramen magnum. Chiari malformations are almost always present at birth (congenital), though symptoms may not appear until later in life. In some cases, the condition is inherited from a biological family and results from a genetic change (mutation). Very rarely, a Chiari malformation can develop in someone who was not born with it. In these cases, the skull or spinal cord may change shape due to a brain tumor, a cyst, hematoma (blood accumulation), hydrocephalus, or intracranial hypertension or pseudotumor cerebri. Chiari malformation may also occur in association with certain underlying health conditions such as Goldenhar syndrome, achondroplasia, spina bifida, connective tissue disorders like Ehlers-Danlos syndrome, or other abnormalities that influence skull or spinal cord development [5-7].

Clinical features (signs and symptoms) 

Clinical manifestations of CM-I range from mild to severe and may vary from person to person. Approximately 30% of these patients may be asymptomatic. The most characteristic symptom is headache, typically located at the back of the head and often triggered or worsened by coughing, sneezing, straining, or bending forward. Many patients also experience neck and shoulder pain due to the compression of the C1-C2 nerves, along with dizziness, vertigo, and difficulties with balance and coordination. Muscle weakness and numbness or tingling in the arms or legs are common, reflecting sensory pathway involvement. Visual symptoms such as double vision, blurred vision, and increased light sensitivity may occur. Hearing disturbances, including tinnitus or hearing loss, are also frequently reported. Additional symptoms include swallowing difficulties, drooling, choking or gagging, and persistent nausea with episodes of vomiting. Some patients develop chronic fatigue, palpitations, or a general feeling of being unwell. 

Sleep disturbances such as insomnia, as well as difficulty breathing during sleep, may also be present. Loss of bladder or bowel control, fainting episodes, and curved spine (scoliosis) may occur, especially when syringomyelia is associated. Because symptoms can fluctuate over time, they may appear in childhood, adolescence, or adulthood and may worsen with activities that increase intracranial pressure. Clinical observations and population-based studies suggest a higher prevalence of CM-I among females compared to males [8-10].

Pathophysiology of CSF flow

CM-I is the most frequently encountered form of Chiari malformations and is defined by the downward displacement of the cerebellar tonsils through the foramen magnum into the upper cervical spinal canal. This structural abnormality alters the relationship between the skull base, brainstem, and cervical cord, leading to the obstruction of CSF flow and mechanical compression of surrounding neural tissues. The resulting disruption in CSF dynamics and pressure regulation contributes to a wide range of symptoms, from occipital headaches and neck pain to sensory disturbances, motor weakness, and, in some cases, the development of syringomyelia [11-13].

**Figure 2:**
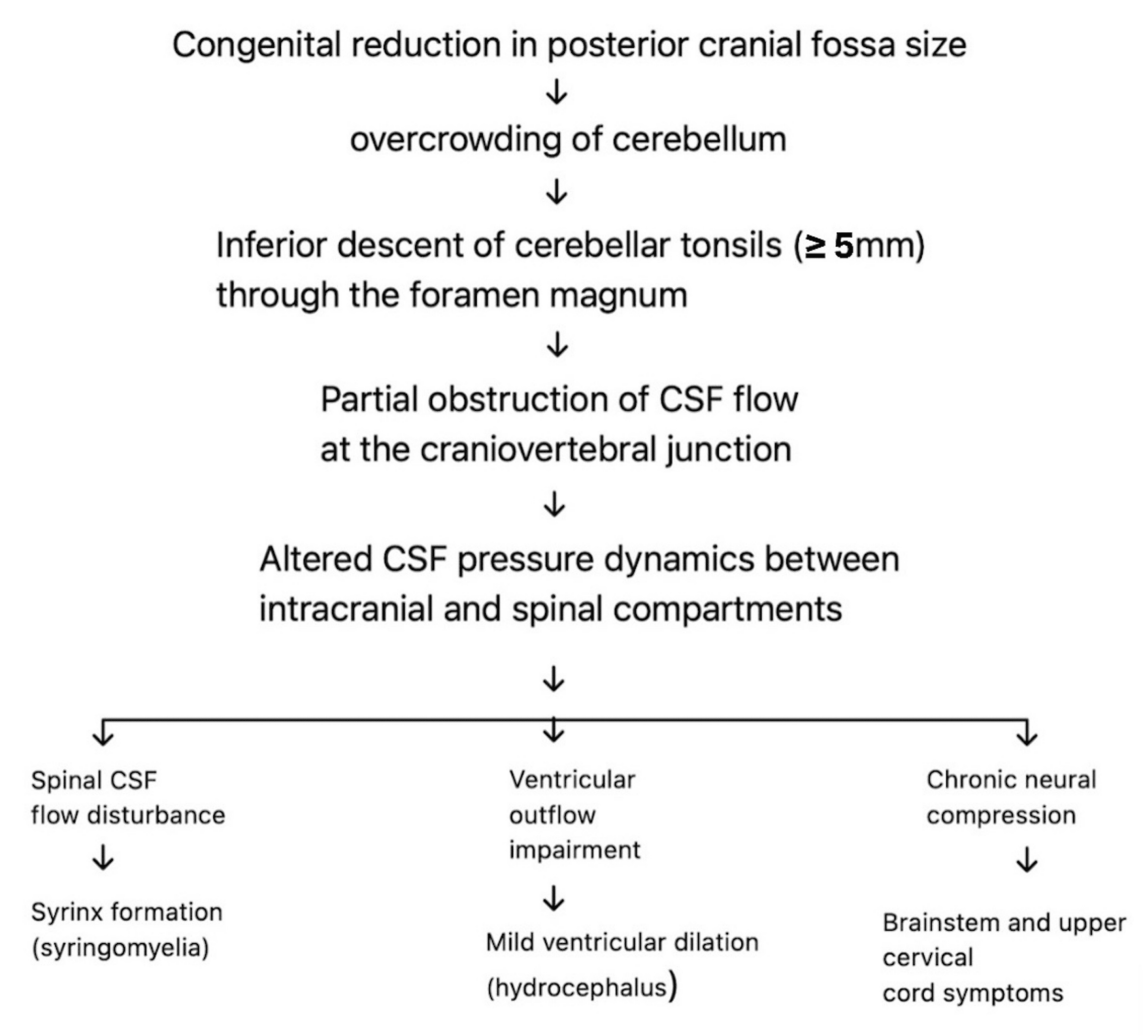
Original flowchart illustrating the pathophysiological mechanisms of CM-I. The diagram highlights cerebellar tonsillar descent ≥5 mm through the foramen magnum, resulting in the partial obstruction of CSF flow at the craniocervical junction and altered CSF pressure dynamics between intracranial and spinal compartments. These changes may contribute to chronic neural compression with brainstem and upper cervical spinal cord symptoms, as well as spinal CSF disturbances leading to syringomyelia. Ventricular dilation and hydrocephalus are shown as secondary findings and are more commonly associated with acquired Chiari malformations rather than classic CM-I Illustration created by the authors and synthesized from established pathophysiological mechanisms described in the literature [2,6-8,11-15]. CM-I: Chiari malformation type I; CSF: cerebrospinal fluid

Figure 2 summarizes the pathophysiological cascade, demonstrating the relationships between ventricular abnormalities, posterior fossa crowding, CSF obstruction, hindbrain herniation, and subsequent hydrocephalus in Chiari malformation.

Management of CM-I includes both conservative and surgical strategies. Treatment decisions typically depend on symptom severity, radiologic findings, and the presence of associated abnormalities such as syringomyelia. A multidisciplinary approach, incorporating neurology, radiology, neurosurgery, and primary care, optimizes diagnostic accuracy, guides the appropriate selection of imaging studies, and improves patient outcomes. Posterior fossa decompression remains the standard surgical intervention for symptomatic cases, aiming to restore normal CSF flow, reduce neural compression, and prevent the progression of associated complications. Objectives of clinical evaluation for CM-I include identifying the underlying etiology, selecting appropriate diagnostic tests, determining the most suitable management plan, and promoting effective communication and coordination across the healthcare team to enhance long-term outcomes. 

Associated conditions and complications of CM-I

Chiari malformations comprise a spectrum of hindbrain abnormalities that vary in severity, with progressive displacement of neural structures leading to the increasing disruption of CSF circulation. As an example, CM-I is characterized by the isolated descent of the cerebellar tonsils through the foramen magnum and commonly progresses to the development of syringomyelia and hydromyelia. A syrinx is a fluid-filled cavity that can develop within the spine and can also occur when CSF is not flowing properly between your brain and spine, due to impaired CSF flow at the craniocervical junction. Moreover, axons of second-order neurons crossing within the spinothalamic tract are mainly affected, leading to the loss of pain and temperature sensation in the bilateral upper extremities. As accumulation expands, it may damage the anterior horn and eventually lower motor neuron (LMN) findings [14,15].

In contrast, Chiari malformation type II represents a more severe form than CM-I with an extensive hindbrain and brainstem displacement and is mainly associated with hydrocephalus, myelomeningocele, syringomyelia, and tethered cord syndrome, a life-threatening condition that occurs when CSF builds up in your head as a result of ventricular outflow obstruction [16]. Hydrocephalus happens when the CSF cannot drain. As a result, CSF buildup occurs, causing an intracranial pressure inside the skull. 

CSF accumulation can cause damage to your spinal cord and result in symptoms like movement and balance problems, pain, muscle weakness, numbness or tingling, muscle spasms, decreased sensation in hot and cold, and loss of bladder and bowel control. Tethered cord syndrome may also occur. Children born with myelomeningocele (a severe form of spina bifida) frequently develop tethered cord syndrome as they grow [16]. This occurs when their spinal cord attaches to their lower back, and over the course of the syndrome, there can be slow and progressive nerve damage that affects the muscles of their body and legs. It can also affect their bowel and bladder function. In addition, symptoms of Chiari malformation can affect your mood, especially if you experience insomnia or severe headaches. Some people may develop depression as well.

Although CM-I is most commonly identified as an isolated congenital complication, it has also been reported in association with rare syndromic and developmental conditions. Case reports in the literature describe associations between CM-I and disorders affecting skeletal and neurodevelopment, including acromesomelic dwarfism, suggesting a possible shared embryological basis related to posterior fossa development and hindbrain crowding. These observations highlight the heterogeneity of CM-I and support the concept that structural abnormalities beyond tonsillar herniation alone may contribute to disease expression [17].

Diagnostic evaluation 

Diagnosis of Chiari malformation typically begins with a complete physical examination. During the exam, the healthcare provider assesses movement, balance, coordination, and sensation in the hands and feet and may also look for memory problems, learning challenges, and developmental delays in children. To confirm the diagnosis, imaging studies of the brain and spinal cord are ordered. MRI is the gold standard for the diagnostic evaluation of CM-I. Sagittal MRI of the brain and cervical spine allows the precise visualization of the cerebellar tonsils in relation to the foramen magnum, with caudal descent exceeding 5 mm generally considered abnormal and supportive of the diagnosis [2]. Beyond confirming tonsillar herniation, MRI is essential for identifying associated conditions, including syringomyelia and other severe manifestations that require prompt recognition, such as hydrocephalus or extensive brainstem involvement [18-20].

**Figure 3:**
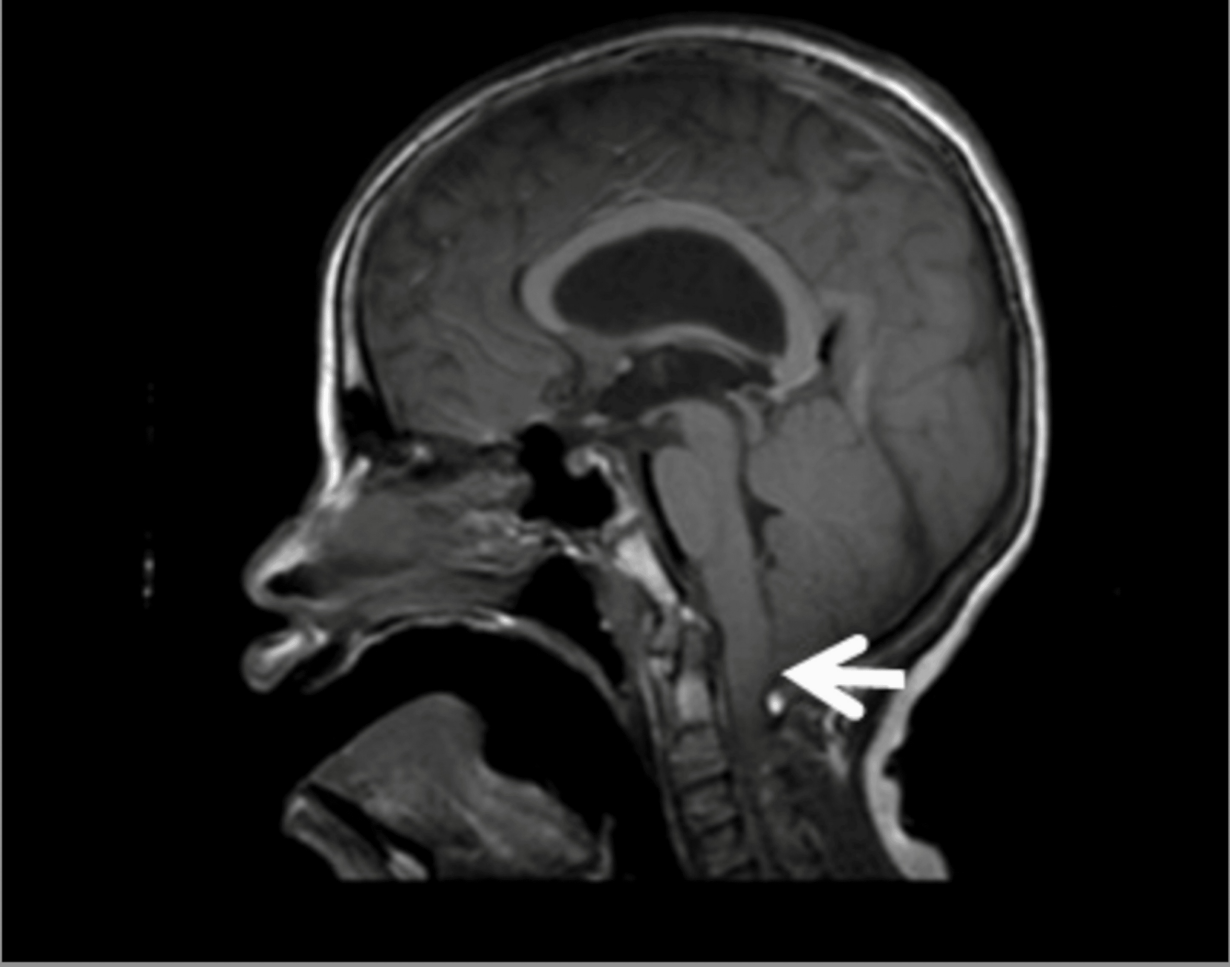
Sagittal MRI demonstrating cerebellar tonsillar herniation (arrow) Image reproduced from Open-i, National Library of Medicine (NIH) [21] (CC BY 3.0 Deed). MRI: magnetic resonance imaging

**Figure 4:**
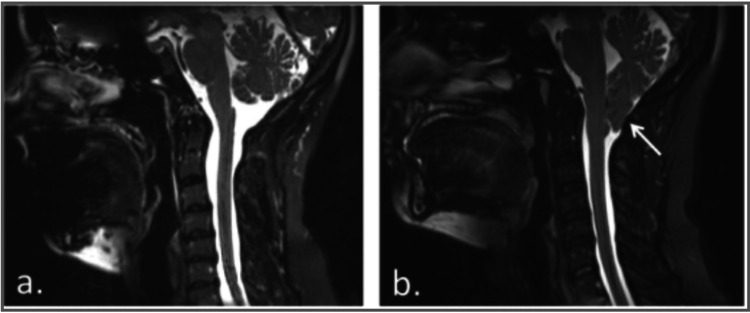
Sagittal T2-weighted MRI showing normal tonsillar position (a) and inferior cerebellar tonsillar herniation consistent with CM-I (b, arrow) Image reproduced from Open-i, National Library of Medicine (NIH) [22] (CC BY 4.0 Deed). MRI: magnetic resonance imaging; CM-I: Chiari malformation type I

Comprehensive imaging of the craniocervical junction and spinal cord, therefore, plays a critical role in assessing disease severity, guiding management decisions, and determining the need for urgent intervention. Representative MRI findings are illustrated in Figures 3-4.

Cine MRI, which is similar to a traditional MRI, allows the visualization of CSF flow around the base of the skull and helps identify flow obstruction. Computed tomography (CT) scans may also be used to detect bony abnormalities of the skull base and spinal column. X-rays can assess bone structure in the skull and neck. In some cases, Chiari malformations are detected on prenatal ultrasounds before birth. Ultrasound uses sound waves to produce real-time images of soft tissues. If a patient does not experience symptoms of Chiari malformation, the diagnosis may be made incidentally while imaging is performed for another unrelated reason. 

Management and treatment

Management of Chiari malformation depends on the severity of symptoms and the degree of neurological involvement. Individuals without symptoms typically do not require treatment, and healthcare providers monitor their condition with periodic clinical evaluations and MRI studies. For mild symptoms such as headache or neck pain, treatment often includes pain medications, particularly nonsteroidal anti-inflammatory drugs (NSAIDs) like ibuprofen or naproxen, as well as muscle relaxants to reduce neck stiffness or spasms. Physical therapy may also be recommended to help manage discomfort and improve function. Patients are advised to avoid activities that increase intracranial pressure, including heavy lifting, straining, and exposure to sneezing triggers such as allergies, as well as chronic coughing. Additional supportive measures may involve massage therapy or using hearing aids or glasses if hearing or vision loss is present.

In more severe or progressive cases, surgical intervention may be necessary. Surgical options include craniectomy, in which the surgeon removes a small portion of the skull to relieve pressure on the brain and improve CSF flow.

In some individuals, surgical intervention is required to manage Chiari malformation. The primary operation performed for CM-I is posterior fossa decompression, a procedure that involves removing a small portion of the skull to relieve pressure on the brain and upper cervical spinal cord. During surgery, the neurosurgeon may also widen the opening of the dura or place a dural graft to increase space and improve CSF flow. In some cases, part of the cerebellar tonsils may be reduced or removed, or a catheter may be inserted to assist CSF drainage. A C1 laminectomy is commonly performed to create additional space [13,23-25].

**Figure 5:**
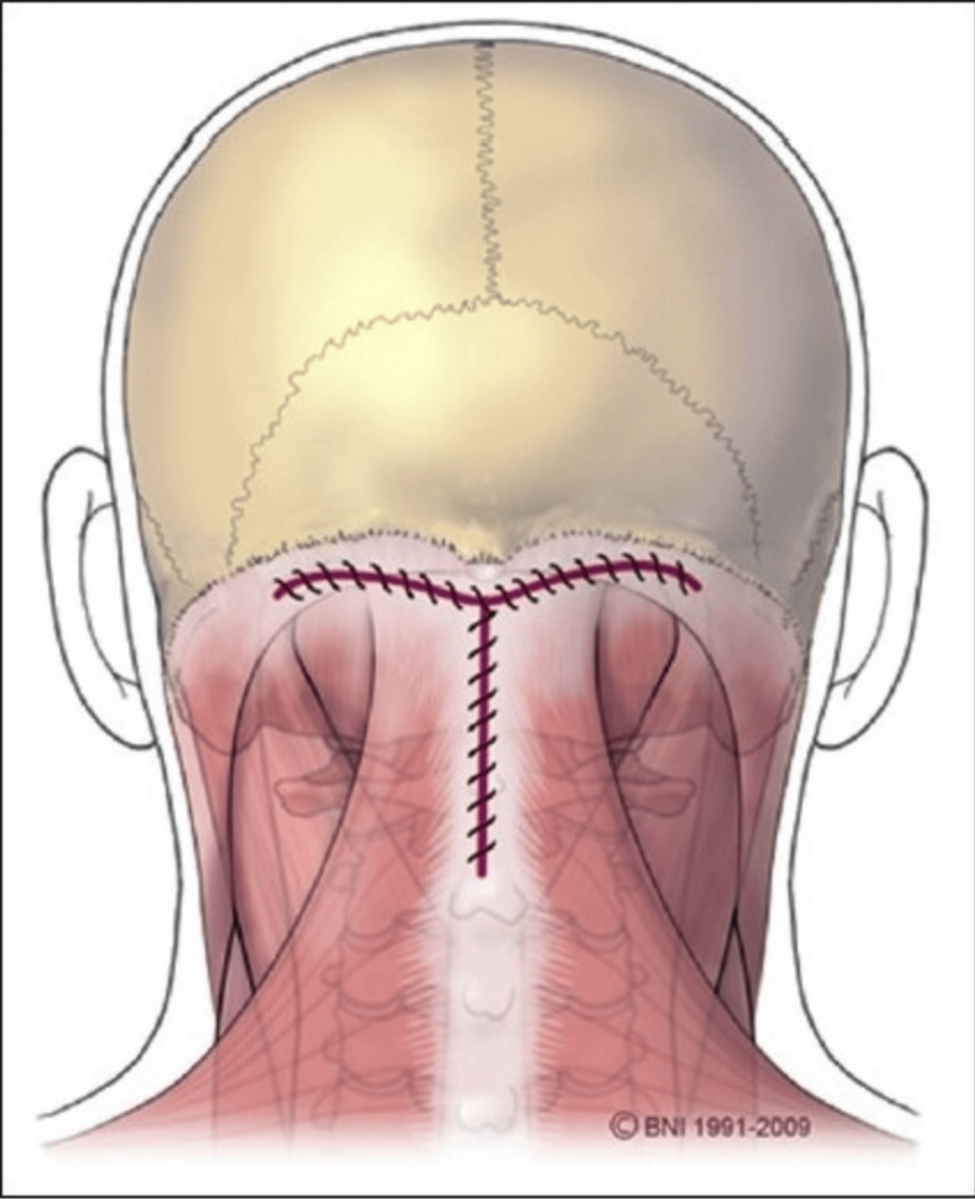
Schematic illustration of a T-shaped fascial incision used in posterior fossa decompression surgery Image reproduced from Open-i, National Library of Medicine (NIH) [26] (CC BY 2.0 Deed).

The overall goal of these surgical techniques is to decompress the brainstem and spinal cord, restore normal CSF circulation, and prevent further neurological impairment. When hydrocephalus is present, a shunt may be placed to divert excess CSF away from the brain and spinal canal to another part of the body. Collectively, these procedures aim to decompress the brainstem and spinal cord, restore CSF flow, improve neurological function, and prevent further deterioration. A schematic illustration of posterior fossa decompression using a representative dural opening is shown in Figure 5.

Prevention of CM-I 

Because CM-I is primarily a congenital structural condition, there are no known methods to prevent its development before birth. However, early recognition and monitoring can help prevent complications by allowing timely intervention. Preventive strategies focus on reducing symptom progression, which includes avoiding activities that increase intracranial pressure, such as heavy lifting, straining, or chronic coughing, and managing contributing factors like allergies that may trigger sneezing. Regular follow-up with healthcare providers and early evaluation of new or worsening symptoms play an important role in preventing long-term neurological impairment.

Prognosis and outlook 

**Figure 6:**
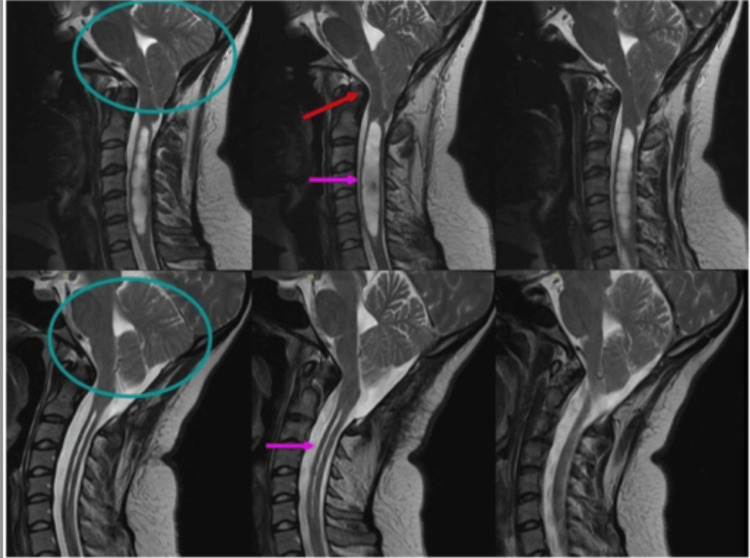
Sagittal T2-weighted MRI demonstrating CM-I before and after surgical decompression. Preoperative images show cerebellar tonsillar herniation with associated cervicomedullary compression, while postoperative images demonstrate the restoration of CSF flow and decompression at the foramen magnum Image reproduced from Open-i, National Library of Medicine (NIH) [29] (CC BY 4.0 Deed). MRI: magnetic resonance imaging; CM-I: Chiari malformation type I; CSF: cerebrospinal fluid

The prognosis for Chiari malformation varies according to the severity of the condition, the presence of associated abnormalities such as syringomyelia, and the timing of treatment. Surgical outcomes are generally favorable, with studies showing that approximately 73% of patients experience improvement within the first postoperative year [27] and up to 79% show sustained improvement at one to three years of follow-up. Posterior fossa decompression leads to symptom relief in nearly two-thirds of patients, particularly those without syringomyelia. When a syrinx is present, it may shrink or resolve following surgical treatment or appropriate management. Headache symptoms often improve significantly, with some reports indicating 80-90% relief after medication or surgery. Radiological pre- and postoperative findings in Figure 6 illustrate the anatomical and physiological changes associated with CM-I and its surgical management [28].

**Figure 7:**
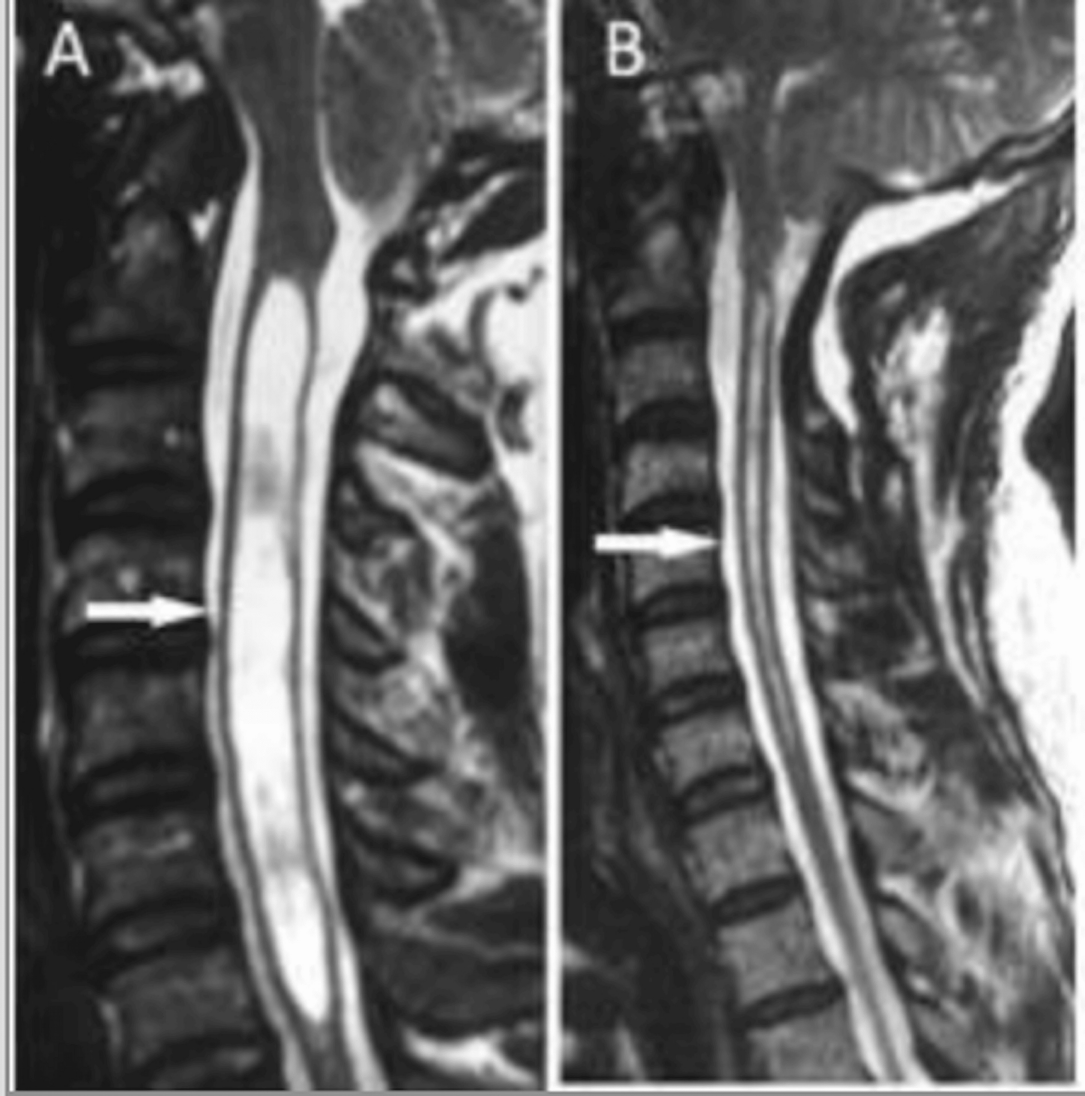
Pre- and postoperative sagittal T2-weighted MRI in a patient with CM-I and syringomyelia. (A) Preoperative image showing a prominent syrinx (arrow). (B) Postoperative image obtained 12 months after surgery showing reduction in syrinx size and restoration of CSF flow Image reproduced from Open-i, National Library of Medicine (NIH) [30] (CC BY 2.0 Deed). MRI: magnetic resonance imaging; CM-I: Chiari malformation type I; CSF: cerebrospinal fluid

As a result, radiological improvement following posterior fossa decompression is illustrated in Figure 7.

Although Chiari malformation can be serious in certain cases, many individuals remain stable for years, and symptoms may not appear until adolescence or adulthood when imaging is performed for another reason. There is no definitive cure, but treatment can reduce symptoms, prevent life-threatening complications, and substantially improve quality of life. Early diagnosis and intervention are associated with better long-term outcomes, and ongoing follow-up with healthcare providers is essential to monitor symptoms and guide individualized care.

Advancing integrated healthcare team efficiency 

Optimal management of CM-I requires a coordinated interprofessional team that may include neurologists, pediatricians, neurosurgeons, nurses, social workers, and physical therapists. Individuals with CM-I often experience a wide range of physical and functional limitations, such as difficulties with swallowing, speech, or gait, making comprehensive and collaborative care essential. Although the overall prognosis is generally favorable, timely recognition and appropriate evaluation, whether for conservative or surgical treatment, are critical to preventing long-term deterioration.

A team-based approach promotes patient-centered care, enhances safety, and improves clinical outcomes. Effective management relies on regular communication among specialists, interdisciplinary case discussions, and shared decision-making to incorporate perspectives from multiple fields. Treatment plans must be holistic, addressing not only neurological deficits but also psychological and social needs. Long-term management includes ongoing monitoring, consistent follow-up, and modifications to treatment strategies as symptoms evolve.

Care coordination further strengthens outcomes by prioritizing patient preferences, involving them in decisions about their care, and streamlining referrals between healthcare providers. Efficient information-sharing systems ensure that all team members have access to accurate and timely patient data. By integrating these strategies and fostering strong interprofessional collaboration, healthcare teams create a supportive environment that promotes positive outcomes, improves patient safety, and effectively meets the complex needs of individuals with CM-I [31].

Discussion

CM-I represents a complex neuroanatomical condition characterized by impaired CSF dynamics, neural compression, and a broad spectrum of clinical manifestations. Although the underlying pathology is well described, significant variability exists in symptom severity, timing of presentation, and radiologic findings, making clinical management challenging. Early recognition remains essential, as timely evaluation allows clinicians to distinguish CM-I from other causes of posterior fossa crowding, including idiopathic intracranial hypertension, craniocervical instability, or incidental tonsillar ectopia. This differentiation is crucial because radiologic tonsillar descent alone does not always correlate with symptom burden and clinical decision-making must integrate both imaging and patient-reported symptoms.

Beyond anatomical explanations, recent studies have begun to explore whether developmental and genetic factors may contribute to the variability seen in CM-I. Some research has examined genes involved in bone development and cranial structure, suggesting that its alterations or constriction is noted to be the primary cause of CM-I. However, findings across studies remain inconsistent. While a few reports propose possible links between CM-I and bone-related genetic pathways, others have not demonstrated a clear relationship between bone mineral density (BMD) and disease expression. These mixed results reinforce the idea that CM-I is a multifactorial condition, in which genetic, developmental, and anatomical factors likely interact rather than act independently. Some research found a relationship between bone-related genes and CM-I due to certain genes that have been associated with craniofacial development [32].

Advances in neuroimaging, particularly MRI and cine MRI, have improved the understanding of CSF flow disturbances and provided a stronger foundation for predicting surgical outcomes. Nonetheless, no universal criteria currently exist to guide the selection of patients who will benefit most from operative intervention. While posterior fossa decompression remains the standard of care for symptomatic individuals, the degree of decompression, the use of duraplasty, and whether tonsillar reduction should be performed vary widely across institutions. Comparative studies have shown generally favorable results, with most patients achieving symptom improvement; however, postoperative complications such as CSF leakage, pseudomeningocele formation, or meningitis highlight the need for careful surgical planning and individualized risk assessment.

Syringomyelia remains an important determinant of prognosis and treatment strategy. Although decompression frequently leads to syrinx reduction, not all patients experience complete resolution, suggesting that factors beyond mechanical CSF obstruction may contribute to syrinx pathophysiology. Ongoing research is needed to clarify the interplay between spinal canal compliance, CSF pulsatility, and neural tissue biomechanics. Furthermore, long-term outcomes vary, particularly among individuals who present in adulthood, emphasizing the importance of continuous monitoring and multidisciplinary care.

Interprofessional collaboration significantly enhances patient outcomes. Coordinated contributions from neurosurgeons, neurologists, rehabilitation specialists, and primary care providers support comprehensive, patient-centered treatment plans. Beyond acute management, long-term strategies should address psychosocial considerations, rehabilitation needs, and education on activity modification to prevent symptom exacerbation. As research continues to evolve, future studies should aim to refine diagnostic criteria, standardize surgical approaches, and identify biomarkers that predict disease progression or surgical success.

Overall, these findings highlight the need of more integrated approach to CM-I that considers anatomical, developmental, and genetic influences when interpreting clinical and imaging findings. CM-I has a generally favorable prognosis when appropriately managed, and ongoing investigation and multidisciplinary care remain central to improving quality of life and long-term neurological outcomes for affected individuals.

Limitations

This review is limited by the variability and quality of available studies on CM-I. Most research consists of observational data rather than randomized trials, which may introduce selection bias. Differences in diagnostic criteria, imaging techniques, and surgical approaches across studies make direct comparisons difficult. Additionally, long-term outcome data, particularly regarding CSF flow dynamics and postoperative improvement, remain inconsistent. These limitations may affect the generalizability of the conclusions presented in this review.

Another limitation of this review is the reliance on heterogeneous study designs, including retrospective analyses, case series, and narrative reviews, which limits the ability to draw definitive causal conclusions. Variability in imaging techniques, diagnostic thresholds for tonsillar herniation, and definitions of syringomyelia across studies further complicates the direct comparison of outcomes. Additionally, publication bias and the relative scarcity of large prospective or randomized studies may influence the strength and generalizability of the findings.

## Conclusions

CM-I is a complex neurological disorder caused by the downward displacement of the cerebellar tonsils through the foramen magnum, leading to impaired CSF flow and a range of clinical symptoms. Understanding its pathophysiology, associated conditions such as syringomyelia, and broad clinical variability is essential for accurate diagnosis and management. Early recognition, appropriate imaging, and timely intervention, whether conservative or surgical, are critical for preventing complications and optimizing long-term outcomes. MRI, including cine MRI, remains essential for diagnosis, assessment of CSF flow abnormalities, and surgical planning. Surgical decompression is the primary treatment for symptomatic patients, with outcomes influenced by anatomical factors, the presence of syringomyelia, and variations in surgical technique. Despite advances in imaging and management, significant variability persists in diagnostic criteria and treatment strategies. Future prospective studies with standardized imaging protocols and outcome measures are needed to improve patient selection, optimize surgical approaches, enhance long-term outcomes, and improve quality of life.
